# Entry skin dose reduction in an inline MRI‐linac using an electron contamination deflector coupled with a helium volume

**DOI:** 10.1002/mp.17923

**Published:** 2025-07-15

**Authors:** Madiha Tai, Jarrad Begg, Elizabeth Patterson, Peter E. Metcalfe, Anatoly Rosenfeld, Bradley M. Oborn

**Affiliations:** ^1^ Centre for Medical Radiation Physics University of Wollongong Wollongong New South Wales Australia; ^2^ GenesisCare St Vincent's Hospital Darlinghurst New South Wales Australia; ^3^ Department of Medical Physics Liverpool and Macarthur Cancer Therapy Centre Liverpool New South Wales Australia; ^4^ Ingham Institute for Applied Medical Research Liverpool New South Wales Australia; ^5^ South Western Sydney Clinical School University of New South Wales Liverpool New South Wales Australia; ^6^ GenesisCare Frankston Private Hospital Frankston Victoria Australia; ^7^ Institute of Radiooncology ‐ OncoRay Helmholtz‐Zentrum Dresden‐Rossendorf Dresden Germany; ^8^ Illawarra Cancer Care Centre Wollongong Hospital Wollongong New South Wales Australia

**Keywords:** inline, MO*Skin*™, MRI‐Linac, skin dosimetry

## Abstract

**Background:**

A unique feature of inline MRI‐linac prototype systems is the changes to entry surface or skin doses. For the high field (1 T) Australian MRI‐linac prototype, a strong surface dose increase is observed. This is a by‐product of secondary electron contamination being focused down the MRI bore due to the magnetic field being parallel with the x‐ray beam direction. It has been anticipated that future clinical treatments will utilize a 2 cm thick water equivalent bolus layer in air above the patient's skin to absorb the electron contamination that causes the large dose hot spot. However, this will reduce the maximum dose rate of the beam and negate the skin dose‐sparing effect typical of MV x‐ray beams.

**Purpose:**

To explore through Monte Carlo (MC) modeling and experimental (Exp) measurements, the skin dose changes when employing a small bespoke permanent magnet electron contamination deflector (ECD) in combination with a helium gas volume (HV) located in the MRI bore. The ECD will purge electron contamination from the beam before it reaches the MRI bore, while the HV minimizes the reintroduction of the contamination above the patient.

**Methods:**

3D magnetic field models of the Australian MRI‐linac coupled with and without the ECD were created in COMSOL Multiphysics, and the magnetic field maps were imported into Geant4 MC simulations. The Geant4 simulations included the 6 MV linac, multileaf collimator (MLC), ECD, HV and a 30×30×30 ${\mathrm{cm}}^{3}$ water phantom positioned at the MRI isocenter. Field sizes of 2.4×2.4 ${\mathrm{cm}}^{2}$, 7.2×7.2 ${\mathrm{cm}}^{2}$, and 12.4×12.4 ${\mathrm{cm}}^{2}$ were simulated and surface skin doses were calculated at 70μm depth. Simulations were performed with and without the MRI field, ECD, and HV to characterize the changes introduced by each component. Exp measurements were also performed using real prototypes of the ECD and HV in the identical environment in order to validate our simulation results. Dose measurements were conducted using a MO*Skin*™ detector and Gafchromic^®^ EBT3 films.

**Results:**

The skin dose hot spot maximum is measured to be 122.5% (2.4×2.4 ${\mathrm{cm}}^{2}$), 194.8% (7.2×7.2 ${\mathrm{cm}}^{2}$), and 260.5% (12.4×12.4 ${\mathrm{cm}}^{2}$) of the dose at $d_{max}$ (1.5 cm depth) with no magnetic field (MC MRI off). Introducing the ECD reduced these values by around 30%. As expected, the reintroduction of electron contamination below the ECD was observed due to the beam requiring transport through another 120 m of air before reaching the phantom surface. Finally, including the HV further reduced the skin dose hot spots by approximately 60% of their original values. The latter results also demonstrated a considerable reduction in the cross‐sectional area of the skin dose hot spot.

**Conclusions:**

The inclusion of an ECD coupled with a HV in an inline MRI‐linac prototype system has significantly reduced the intensity of the skin dose increases. It appears to be superior solution over a generic 2 cm water bolus for all field sizes and also will retain some skin dose‐sparing effect. The methods used in this work are expected to be feasible with other inline MRI‐linac designs.

## 1 INTRODUCTION

The integration of magnetic resonance imaging (MRI) with linear accelerators (linacs) has advanced the field of image‐guided radiation therapy (IGRT). The advantages include improved positional verification prior to treatment, including the ability to rescan and replan patients due to patient and tumor changes, and real‐time monitoring of the target to assess internal movement during treatment. This results in a reduction in setup errors and improves tumor control probability.^1^ Along with the real‐time visualization of the target, the MRI‐linac technology also offers superior soft tissue contrast and improved accuracy of tumor and organ delineations. However, the presence of the strong magnetic field coupled with the radiation beam brings forward unique challenges that impact dosimetry and the design of the system. At present, the Elekta Unity and the ViewRay MRIdian machines are in clinical use.^2^, ^3^

In recent decades, various studies have demonstrated changes in dose distribution due to the presence of the magnetic field. Although the photon beam remains unaffected, the trajectory of secondary electrons is modified by the Lorentz force, which impacts the overall dose distribution. In the presence of the transverse magnetic field, the changes in dose distribution are highly pronounced on entry and exit surfaces, as well as in the medium with different densities.^4^, ^5^, ^6^, ^7^ Previous studies have also shown that the presence of the transverse magnetic field can change the response of several solid‐state detectors and ionization chambers.^8^, ^9^, ^10^ These changes are angular and magnetic field strength dependant, therefore; specific orientations and correction factors need to be applied to ensure accurate dosimetry.^11^, ^12^, ^13^, ^14^ The skin dose distribution on the entrance and exit surfaces is also found to be altered in the presence of the transverse magnetic field.^15^, ^16^ Thus, the overall changes in beam characteristics in MRI‐linac can make dosimetry more complex. On the other hand, a longitudinal magnetic field, relative to the primary beam direction, is reported to have minimal impact on dose distribution and overall beam characteristics of megavoltage (MV) photon beam.^17^, ^18^, ^19^ Monte Carlo (MC) studies have shown that in the presence of the inline magnetic field, electrons converge along the beam trajectories due to the Lorentz force acting inline relative to the primary beam direction.^20^ These electrons are found to be more focused along the direction of the beam, which reduces lateral scattering and narrows down penumbral widths and consequently, conformal dose distribution at depths within homogeneous medium can be achieved.^21^, ^22^, ^23^, ^24^

For a MV photon beam, electron contamination largely contributes to the dose in the build‐up region.^25^, ^26^ It arises usually from the linac head, flattening filter, beam modifiers, collimation devices and in the air space between linac head and patient surface^27^, ^28^, ^29^, ^30^, ^31^ and is also dependent on field sizes and source‐to‐surface distance (SSD).^30^, ^32^, ^33^, ^34^, ^35^ In the presence of the inline magnetic field, electron contamination, instead of scattering laterally, is highly focused within the beam and consequently, skin dose increases.^36^, ^37^ In a MC study conducted by Oborn et al (2012), the skin dose increases significantly in the presence of the inline magnetic field and is also dependent on the SSD, field sizes, and beam collimation arrangement.^36^ This study was further extended by Oborn et al (2014) for a 1T open bore MRI‐linac design and skin dose values were again found to be of similar magnitude.^38^ Experimentally measured skin dose values for a similar inline MRI‐Linac design have also shown significant differences in the presence of 0 T, 1 T and 1.5 T fields.^39^, ^40^, ^41^, ^42^

In a recent study, our research group has performed MC simulations for the modeling of skin dose for Australian MRI‐linac prototype and has reported surface dose hot spots at 70 μm depth as a percentage of the dose at $d_{max}$(1.5 cm depth).^42^ These skin dose values are further compared with the experimental (Exp) study performed for the Australian MRI‐linac using a MO*Skin*™ detector system^41^ and are within ±10% agreement. These results also highlight the focusing effect of electron contamination toward the beam's central axis and its contribution toward the surface dose. The surface dose values measured in the simulations are also high for the large field sizes due to contamination being focused in the presence of the magnetic field.^42^

Several methods have been investigated to reduce surface dose caused by electron contamination. Some studies have explored the use of beam spoiler/bolus and filters, but some of these methods reintroduced electrons within the material, which resulted in an increase in surface dose within the phantom.^43^, ^44^ An off‐axis irradiation technique has also been investigated in one experiment to minimize the surface dose caused by electron contamination. However, off‐axis irradiation reduces the available treatment field size and MRI images have larger geometric distortions and non‐linearities at increasing distances from the MRI isocenter, which reduces the clinical utility of the MRI‐linac.^41^, ^45^ An alternative method has been suggested which involves using a permanent magnet deflector to purge electron contamination from the x‐ray beam,^46^ as well as a helium volume to reduce air‐generated electron contamination.^28^, ^46^ A MC study was conducted by Oborn et al (2014)^38^ to investigate the effectiveness of using a combination of these two methods for a 1 T inline MRI‐linac. The results showed significant reduction in surface dose and the size of the hot spot.^38^ The aim of this study was to explore the performance of these two electron contamination reduction techniques through both simulations and Exp measurements. To achieve this, the existing MC (Geant4) and finite element models (COMSOL Multiphysics) of the Australian MRI‐linac system,^41^, ^42^ were extended to include an electron contamination deflector (ECD) and a helium volume (HV). Although the actual designs of the ECD and HV are suited to this MRI‐linac prototype, the methods are applicable to any inline MRI‐linac prototype system. In order to validate the simulation predictions, Exp measurements were also performed using real prototypes of the ECD and HV. Dose measurements for the experiments were conducted using the MO*Skin*™ detector because of its suitability for in vivo skin dosimetry.^47^ Gafchromic^®^ EBT3 films were also used at varying distances from the source to qualitatively analyze the changes in the dose hot spot with respect to the distance.

## 2 MATERIALS AND METHODS

**FIGURE 1:**
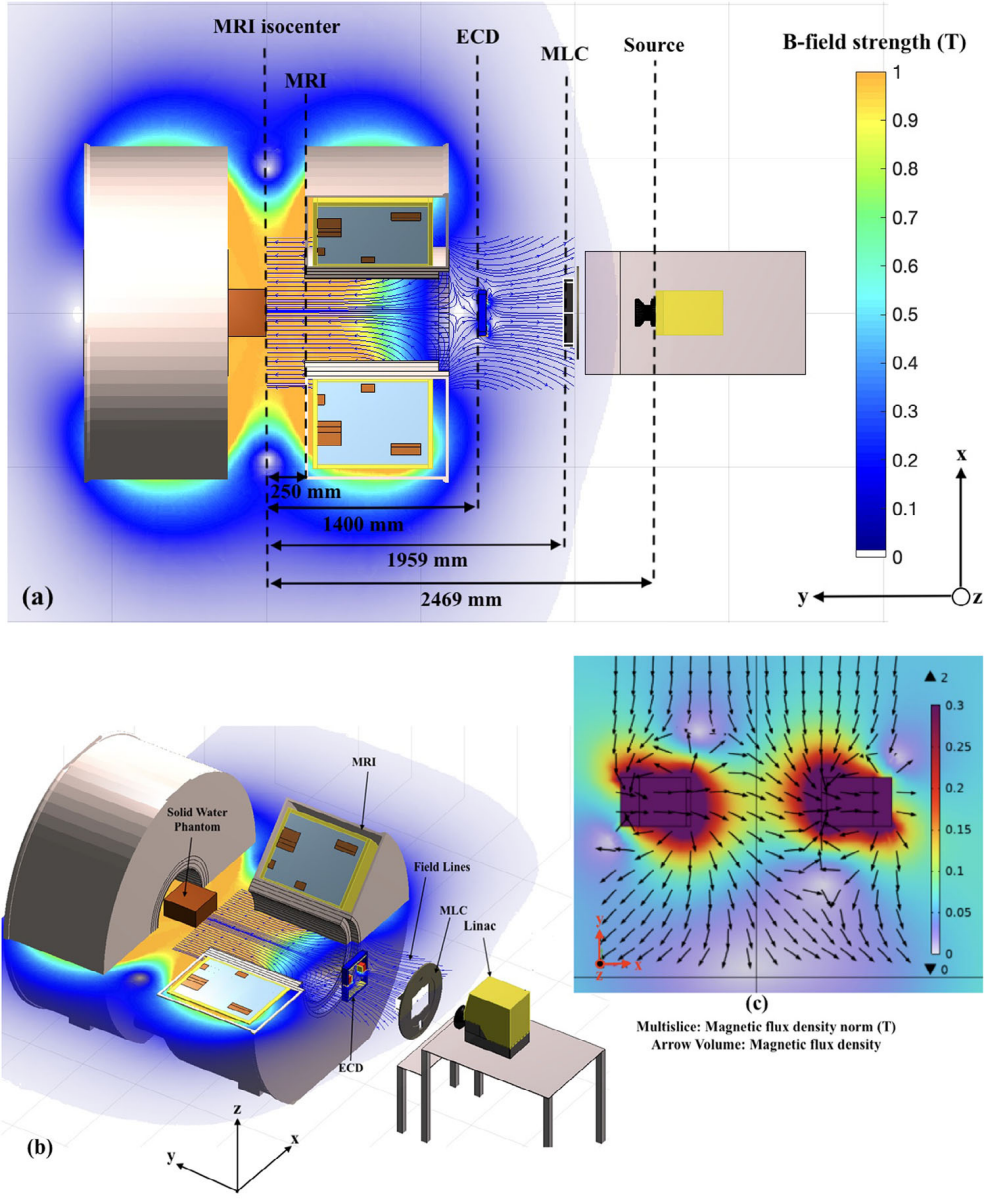
Schematic diagram of the Australian MRI‐linac setup at the Ingham Institute is shown in (a) and (b). The setup includes the split‐bore MRI, linac, MLC and solid water phantom. It also shows the visualization of the ECD prototype positioned 1400 mm distal from the phantom surface. The magnetic field intensity (T) is superimposed using a colormap with a transparency inversely proportional to the magnitude, that is, the white background is observed in the low field zones. Part (c) displays the magnetic flux surrounding the ECD as seen from topview. ECD, electron contamination deflector; MLC, multileaf collimator.

The Australian MRI‐linac system is currently under research use at the Ingham Institute for Applied Medical Research, Liverpool, Australia. The MRI‐linac prototype consists of a Varian flattening filter‐free (FFF) linear accelerator (Varex UT, United States) coupled with a custom‐designed 1 T split bore MRI scanner (Agilent Technologies Oxford, United Kingdom). The linac is mounted on a rail system whereby the distance between source to MRI isocenter can be varied between 1800 to 3200 mm. The linac produces a primary 6 MV x‐ray beam which is collimated by a Millenium 120 leaf (Varian, Palo Alto, CA, USA) multileaf collimator (MLC) located at 510 mm downstream of the x‐ray source. A schematic diagram of the Australian MRI‐linac system is shown in Figure 1. All work has been conducted at an SSD of 2469 mm which is the intended setup for future clinical trials.

### 2.1 Monte Carlo modeling and simulations

The modeling and simulations have been performed in two stages using two software packages. In the first stage, the magnetic fields produced by the MRI and the ECD are simulated using finite element methods. In the second stage, the radiation beam transport is simulated using MC methods. These two stages are described below.

#### 2.1.1 Magnetic field model of the MRI scanner

The 3D magnetic field map of the 1T Australian MRI‐linac has previously been modeled and benchmarked^38^ using COMSOL Multiphysics v4.4.^48^ This MRI only model consists entirely of the superconducting MRI source coils and accurately details the magnetic field distribution over a volume that encompasses the MRI and nearby linac. This first simulation provides the 3D magnetic field map data for later import into the Geant4 MC simulations of the skin dose with the MRI field only, i.e., the ECD is not present.

#### 2.1.2 Magnetic field model of the ECD

**FIGURE 2:**
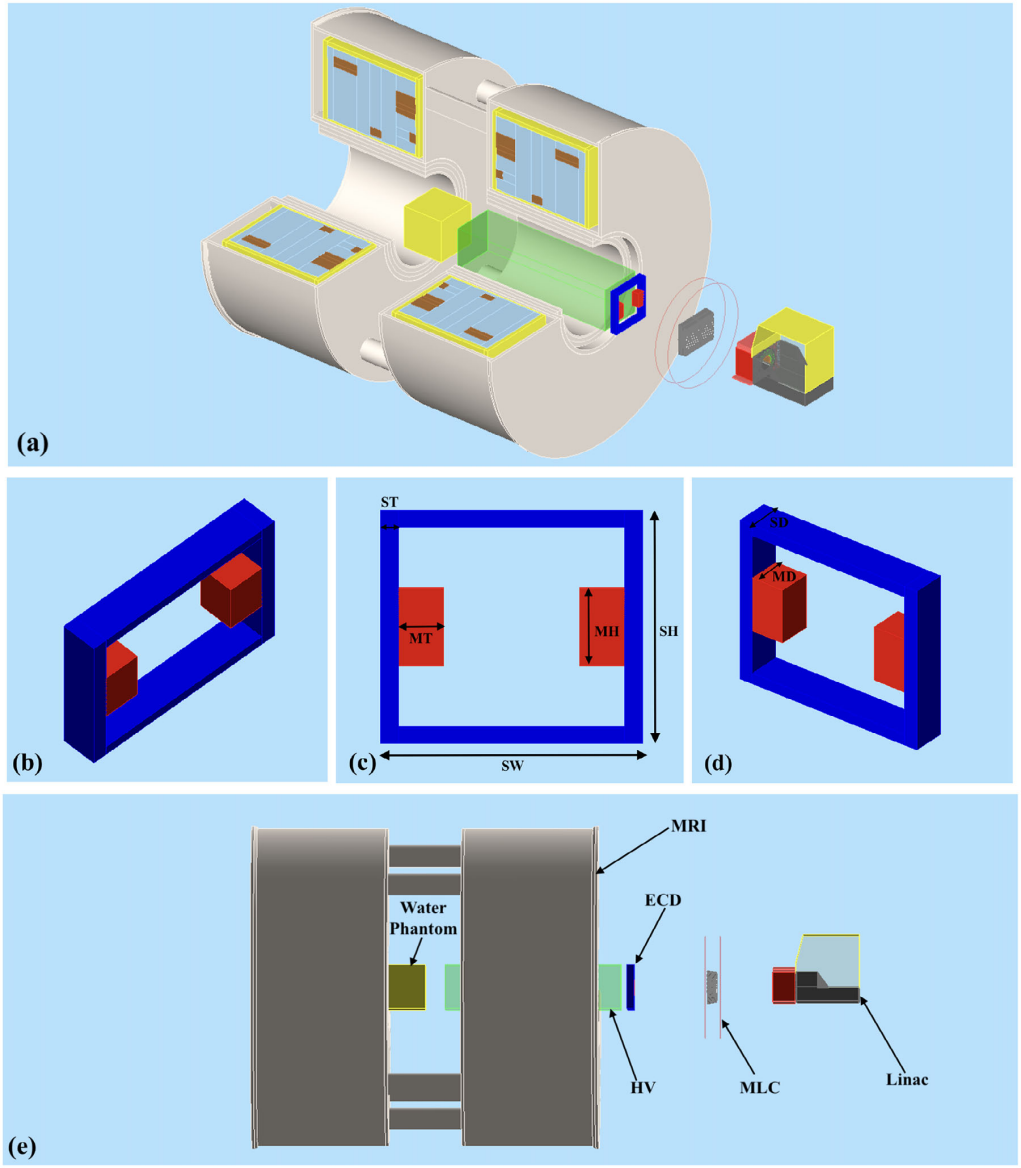
Geant4 MC setup for the simulations of the Australian MRI‐Linac system. The top (a) and bottom (e) view shows the split‐bore MRI‐linac, MLC, water phantom, ECD and HV. The center images (b), (c) and (d) show the schematic of the ECD having Magnet Thickness (MT) = 5 cm, Magnet Height (MH) = 10 cm, Magnet Depth (MD) and Steel Depth (SD) = 5 cm, Steel Width (SW) = 25 cm, Steel Thickness (ST) = 2 cm and Steel Height (SH) = 20 cm. The helium volume region has dimensions of 30×120×30 cm^3^. MC, Monte Carlo; MLC, multileaf collimator; ECD, electron contamination deflector; HV, helium gas volume.

In the second benchmarking simulation, an accurate model of the permanent magnet‐based ECD was added to the previous benchmarked MRI only COMSOL model. The ECD was positioned 1400 mm distal from the phantom surface. The ECD modeled consisted of two rare earth magnet banks composed of NdFeB and mounted within a plain low‐carbon steel frame, as shown in Figure 2. The magnet banks had a thickness of 5 cm, depth of 5 cm and height of 10 cm, while the steel frame had a cross‐sectional area of 2×5 ${\mathrm{cm}}^{2}$ and held the magnet banks apart with a width of 25 cm to allow large x‐ray field sizes to pass through. The magnetic field value between the magnet poles was 0.13 T. The magnetic field maps, including the ECD, were also imported into the Geant4 MC simulations for the second set of simulations to examine the change in skin doses with the inclusion of the ECD.

#### 2.1.3 Geant4 MC model

The Geant4 MC model of the 1 T Australian MRI system was designed in Geant4 10.06.01 and the simulations were split into two components. The first part comprised of simulating the x‐ray beam production from the linac head to create a collection of phase space files located above the MLC. The second stage simulations comprised of transporting particles from these phase space files through to the phantom for dose calculations at the MRI isocenter. The following sections describe these in more detail.

#### 2.1.4 Linac head simulations

The linac x‐ray head was modeled according to the manufacturers specifications. The model included the x‐ray target (copper), the primary collimator (tungsten), the ionization chamber, and the beam exit window (aluminum). The geometry also included collimator housing, magnetic shielding, and linac cover for completing the model but the addition of these components had no impact on the beam generation or collimation. Each MLC leaf was made up of a tungsten alloy with a default gap of 40 μm. The leaf tips were located at a distance of 1959 mm from the MRI isocenter. For x‐ray production, a total of 200 phase space files were generated that consisted of a 6.0 MeV electron pencil beam striking the target and simulated 1 x 10^8^ primary electrons in each file with 1% Gaussian spread in energy. Each file produced 3.3 x 10^8^ secondary particles which then traveled through the MLC down to the phantom located at the isocenter of the MRI scanner. For the first set of simulations, the phase space files were generated without including the magnetic field data. This was done to simulate particle interactions with no magnetic field (0 T), serving as a baseline for normalizing and comparing skin dose in the presence of the magnetic field. For the phase space files of MRI‐linac, the magnetic field data maps from COMSOL were added in the second and all subsequent sets of simulations. The MLC position was set for nominal field sizes of 1×1 ${\mathrm{cm}}^{2}$, 3×3 ${\mathrm{cm}}^{2}$ and 5×5 ${\mathrm{cm}}^{2}$ at 1000 mm SSD. These field sizes were magnified to 2.4×2.4 ${\mathrm{cm}}^{2}$, 7.2×7.2 ${\mathrm{cm}}^{2}$ and 12.4×12.4 ${\mathrm{cm}}^{2}$, respectively at the standard SSD setup of 2469 mm and were used for all sets of simulations.

#### 2.1.5 Monte Carlo model of the ECD and the helium bag

The ECD geometry was included in the Geant4 model below the MLC with the same geometry and materials simulated in COMSOL and was placed 1400 mm upstream above the phantom surface, which corresponds to just below the MLC, as shown in Figure 2. The HV region was modeled in Geant4 by defining a helium gas volume (HV) with dimensions of 30×120×30 cm^3^, enclosed by a 27 μm thick layer of High‐density polyethylene (HDPE) material on the distal and proximal surface of HV. This thickness was kept as minimal as possible to avoid the generation of secondary electrons. The distal surface of HV was aligned with the upper edge of the cryostat wall and the proximal surface was at 13.5 cm above the MRI isocenter as shown in Figure 2a, e. The distance between the HV distal surface and the ECD was 6 cm. These positions and dimensions were selected by considering the actual MRI‐linac machine and the feasibility of adding these components in the clinical environment.

#### 2.1.6 Phantom dose simulations

For the dose calculations, a water phantom measuring 30×30×30 cm^3^ was used, with its surface positioned at the MRI isocenter. The Gammex Solid Water material was used in Geant4 for the phantom's composition. Two dose‐scoring volumes were used to measure the dose changes within the phantom. The first dose scoring volume, encompassed the entire phantom with each scoring voxel measuring 1×1×1 mm^3^ to measure the depth dose distribution throughout the phantom. The second dose scoring volume with each high resolution scoring voxel measuring 10 μm in the depth direction (*y* axis) and 1 mm×1 mm in the cross‐section direction (*x* and *z* axis), was positioned at first 2 cm of the phantom to measure the entrance surface dose at 70 μm depth. The depth of 70 μm (basal layer) is recommended by the International Commission on Radiological Protection to asses the skin dose.^49^, ^50^ The particle source for these simulations were the phase space files produced from the previous simulations of the linac head.^42^

The first set of MC simulations was performed without magnetic field data (MC MRI off). In the second set of simulations (MC MRI on), the magnetic data field data maps from COMSOL were used. In the third set of simulations (MC MRI on + ECD), the ECD was included in the magnetic data field maps in COMSOL and in the Geant4 geometry. In the fourth set of simulations (MC MRI on + ECD + HV), the same magnetic field data from COMSOL, which accounted for the presence of ECD, was used along with ECD and HV incorporated in the Geant4 geometry. The simulation setup in Geant4 is shown in Figures 1 and 2. The data obtained in the presence of the magnetic field was normalized and compared to the data obtained from the simulations with no magnetic field (MC MRI off). All depth dose curves were normalized at a depth of 10 cm. The PDDs are expressed as the percentage of the maximum dose ($d_{max}$) at 1.5 cm depth for the 0 T measurements (MC MRI off). The total combined uncertainty in the simulated dose was determined to be ±5% at the 70 μm depth for the B = 0 T reference simulations.

### 2.2 Experimental measurements

This study is further validated by the Exp measurements conducted at the Australian MRI‐linac system at the Ingham Institute for Applied Medical Research in Liverpool, Australia. All measurements were performed using a 30×30×30 cm^3^ solid water phantom (Gammex‐RMI, Middleton, WI, USA) placed inside the MRI bore with it's surface positioned at the MRI isocenter. The measurements were performed using the same three field sizes defined at the isocenter of 2.4×2.4 ${\mathrm{cm}}^{2}$, 7.2×7.2 ${\mathrm{cm}}^{2}$ and 12.4×12.4 ${\mathrm{cm}}^{2}$. This corresponded to an SSD of 2469 mm. For our Exp work, the beam portal window (copper radio‐frequency shield layer) was removed to allow for placement of the HV. It is feasible that this window can be moved distal of the ECD to maintain a complete RF shield in the future if the ECD + HV are used clinically.

#### 2.2.1 The prototype of ECD and the Helium bag

**FIGURE 3:**
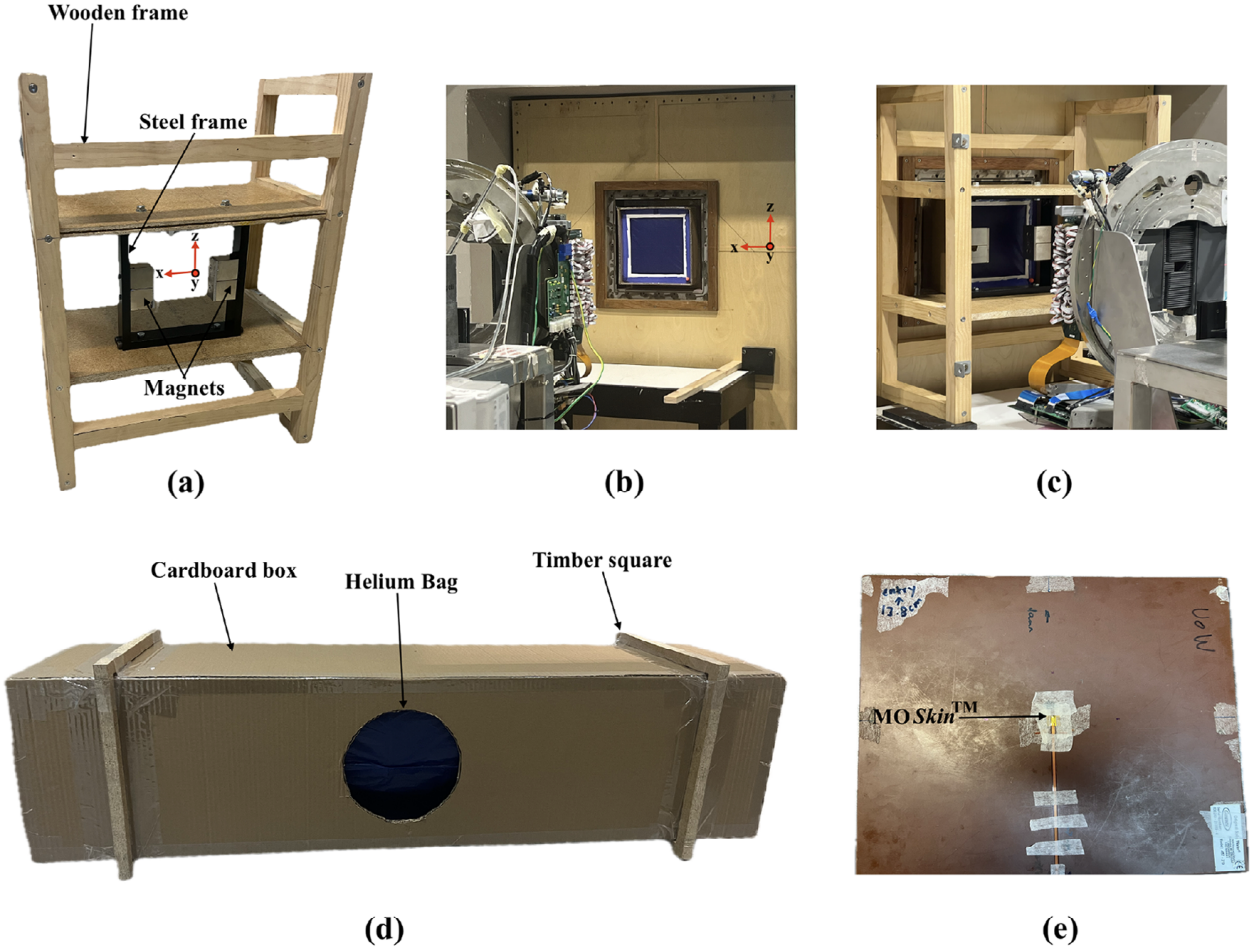
(a) Prototype of the ECD placed within wooden frame, (b) distal surface of HV aligned with the cryostat wall, (c) ECD and HV setup at an SSD of 2469 mm, (d) prototype of HV and (e) MO*Skin*™ solid water phantom. ECD, electron contamination deflector; HV, helium gas volume; SSD, source‐to‐surface distance.

The ECD prototype was constructed using two 4 kg rare earth magnet banks, mounted within a steel frame, as shown in Figure 3a,b. The steel frame, weighing around 11 kg, was designed to support the magnet banks, which were secured to the frame with screws and positioned 25 cm apart, permitting large x‐ray field sizes to pass through. The ECD prototype, designed to match the geometry and dimensions of the Geant4 model, was positioned beneath the MLC at the same location, 1400 mm upstream from the phantom surface. It was enclosed in a robust wooden frame and placed on a wooden benchtop just outside the RF cage wall as shown in Figure 3c,h.

The prototype for the HV was constructed using a 27 μm thick plastic bag enclosed in a cardboard box with internal dimensions of 28.5×28.5×121.0 cm^3^. The cardboard box had open ends to allow the beam to pass through the plastic, and timber squares were used to secure the box, as illustrated in the Figure 3e–g. For the measurements, the bag was filled with helium gas (99.9% purity), reaching a total length of 118.0 cm. The HV was filled without any pressurization or optimization beyond atmospheric pressure as the aim was to simply replace the air with the lower density helium gas. To minimize helium loss and maintain a consistent cross‐sectional area of the helium zone, the gas was introduced on the same day as the measurement. The HV was positioned horizontally along the beam direction (*y*‐axis) such that its proximal and distal surfaces were 13.5 and 131.5 cm from the MRI isocenter respectively. These positions and dimensions were designed in order to replace as much of the beamline air volume as possible with helium, while still allowing a feasible space around the treatment isocenter. With the HV proximal surface being 13.5 cm from isocenter, it would be possible to treat a head/brain tumor with the patient centered around MRI isocenter. For larger treatments, the proximal edge of the HV would need to be moved further from isocenter.

#### 2.2.2 Dosimetry

**FIGURE 4:**
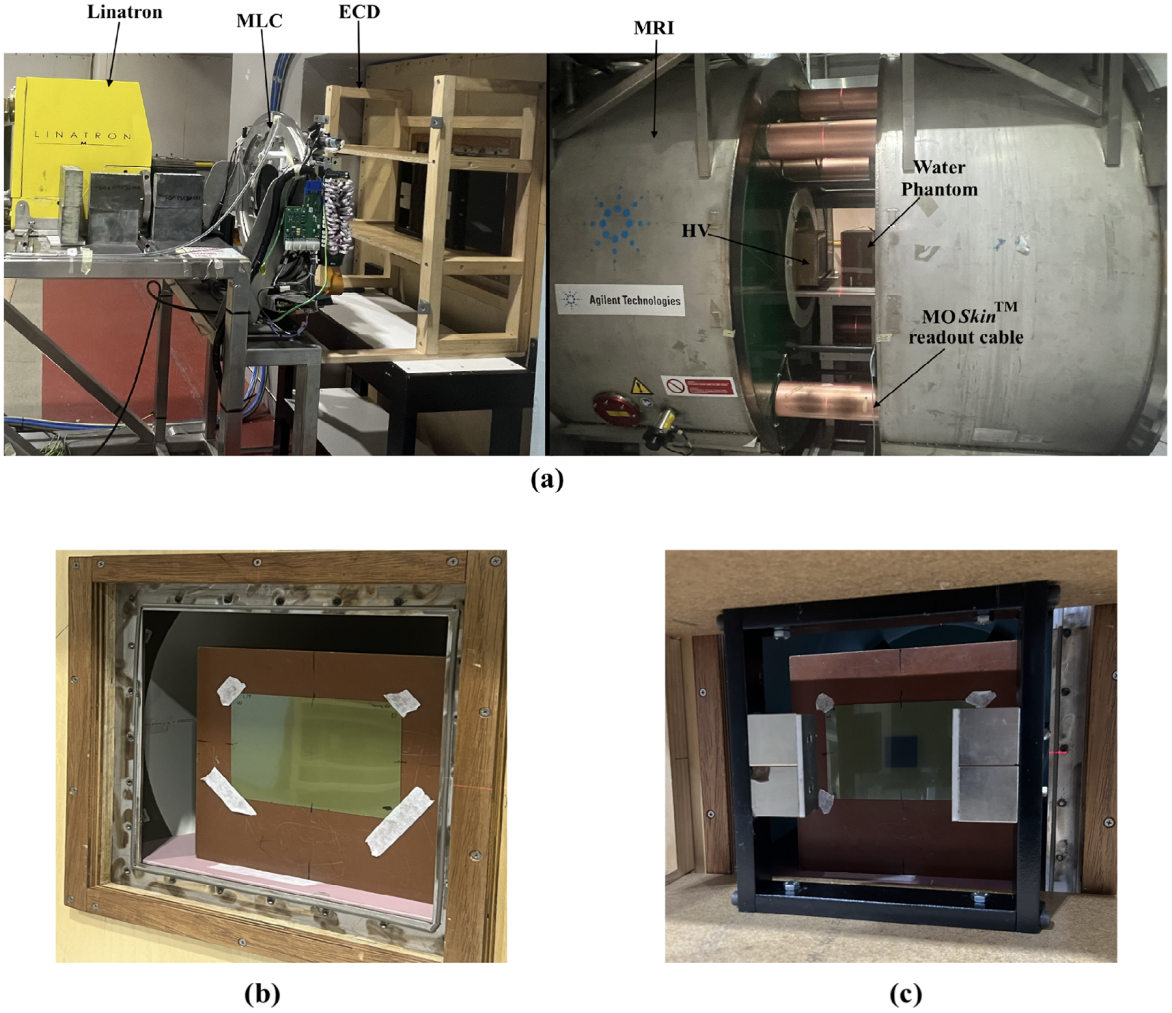
Exp setup at the Australian MRI‐linac system at the Ingham Institute for Applied Medical Research, Liverpool, Australia. (a) The Linatron mounted on a rail system, MLC and prototype of ECD on left and 1 T split bore MRI scanner, prototype of HV and MO*Skin*™ detector attached with 30×30×30 cm^3^ phantom at MRI‐isocentre on right. (b) The Gafchromic^®^ EBT3 film attached to the water phantom placed at 120 cm above MRI isocentre. (c) The Gafchromic^®^ EBT3 film attached to the water phantom placed at 120 cm away from MRI isocentre in the presence of ECD. ECD, electron contamination deflector; Exp, experimental; MLC, multileaf collimator; MRI, magnetic resonance imaging; SSD, source‐to‐surface distance.

The Exp dose measurements used both the MO*Skin*™ detector and Gafchromic^®^ EBT3 films (Ashland, Covington, USA). The MO*Skin*™ is a metal oxide semiconductor field‐effect transistor (MOSFET) detector designed to measure the dose to the basal layer of the skin, with water equivalent depth (WED) of 70 μm. The detector's sensitive volume is approximately 1500 μm ^3^, encapsulated by a polyamide layer with a water‐equivalent thickness of 70 μm. The detector and its readout system were designed and developed by Centre of Medical Radiation Physics (CMRP), University of Wollongong, Australia.^51^ To ensure minimal air gaps, a customized water phantom with a 1 mm recess, as shown in Figure 3e, was used to hold the detector. The measurements were taken using a solid water phantom positioned vertically at a fixed SSD. The detector depth was adjusted incrementally by adding layers of 50 μm polyamide (Kapton) tape to the MO*Skin*™ up to a depth of 0.027 cm. For depths ranging from 0.027 to 10 cm, solid water phantoms of varying thicknesses were placed in front of the detector. The Exp setup is illustrated in Figure 4a. The Gafchromic^®^ EBT3 film was primarily employed for qualitative analysis of the skin dose in MRI‐linac (MRI on) and with ECD (MRI on + ECD) at different distances between the ECD and film toward the MRI isocenter. The MO*Skin*™ with the gate bias +15V during irradiation, was used to obtain high‐resolution, near‐surface dose measurements in MRI‐linac (Exp MRI on), with ECD (Exp MRI on + ECD) and with ECD and HV (Exp MRI on + ECD + HV) at 70 μm depth. For each MO*Skin*™ position, three readings were taken and averaged. The quoted uncertainties in the measurements include both statistical and systematic components. Statistical uncertainty is calculated as the 95% confidence interval, normalized to the $d_{max}$. Systematic uncertainty, estimated at 3.5%, accounts for potential variations in field intensity and detector positioning.

To ensure consistency, all the Exp measurements were also normalized at a reference depth of 10 cm for near 0 T simulations (MC MRI off). The MO*Skin*™ measured PDDs are expressed as the percentage of dose at $d_{max}$ for near 0 T simulations (MC MRI off). The linac output was monitored using a A CC13 chamber (s/n: 15996, IBA Dosimetry GmbH). The reference chamber was placed in the air, positioned between the linac and the MLC within the beam's field so that each MO*Skin*™ measurement could be corrected for variations in the linac output.

For the qualitative analysis of hot spot with respect to the distance, the film was placed at different distances from the source toward the MRI‐isocenter (MRI On). These measurements were repeated with the same setup and distances in the presence of ECD (MRI on + ECD) as shown in Figure 4b,c. At each distance, the film was irradiated for a total of 21 gray (Gy) exposure, using a field size of 7.2×7.2 ${\mathrm{cm}}^{2}$. The film measurements were performed exclusively to evaluate how effectively the ECD reduces the hot spot when placed at different distances; thus, the films were scanned and plotted without any processing

## 3 RESULTS

### 3.1 Electron contamination purging and reduction using the ECD and HV

**FIGURE 5:**
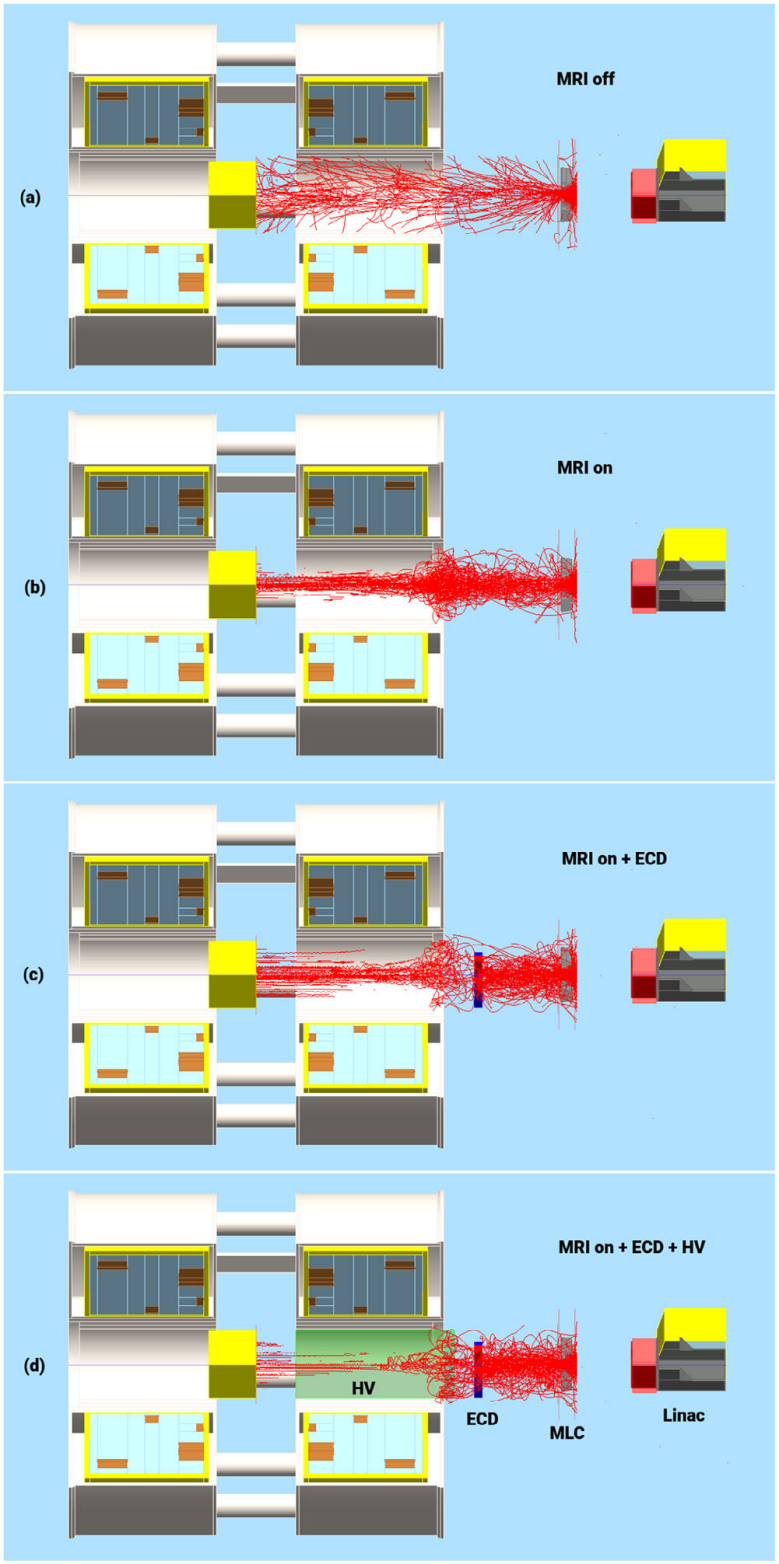
Geant4 MC simulations illustrating the electron contamination paths between the MRI isocenter and MLCs. The magnetic focusing of electrons is evident in images (b), (c), and (d). The addition of the ECD below the MLC disrupts the focusing effect, as seen in (c). Further contamination reduction is seen in (d) with the inclusion of both the HV and ECD. ECD, electron contamination deflector; HV, helium gas volume; MC, Monte Carlo; MLC, multileaf collimator.

**FIGURE 6:**
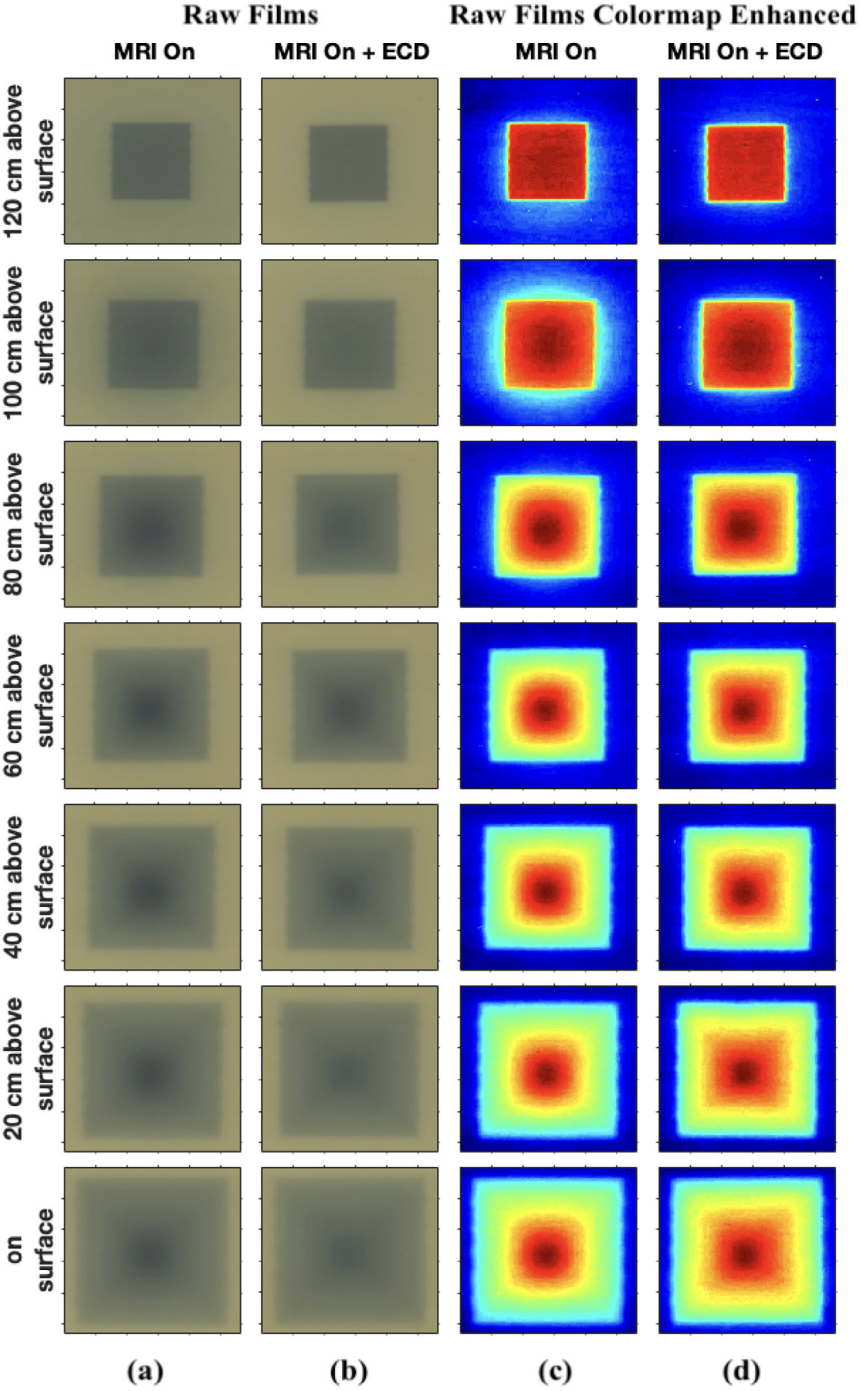
Visualization of the hot spot on the films in the presence of the magnetic field with respect to distance. The presence of the magnetic field causing highly focused hot spot in the center of the beam can be seen in (a) and (c). The inclusion of the ECD below the MLC has reduced the focusing effect in (b) and (d) upto a certain distance. ECD, electron contamination deflector; MLC, multileaf collimator.

A qualitative demonstration of the electron contamination that creates the skin dose hot spot is presented in Figure 5 using screenshots of the Geant4 MC simulations. In part (a) the paths of electron contamination are shown when there is no magnetic field present (MC MRI off). Part (b) represents the case with the MRI on, showing how there is a strong focusing effect toward the beam's central axis (MC MRI on). In part (c), the ECD is included (MC MRI on + ECD), which demonstrates how contamination is purged from the beam as it passes through the local magnetic field of the ECD. Finally part (d) demonstrates the inclusion of the HV (MRI on + ECD + HV), where the reintroduction of the electron contamination is minimized over the last 1200 mm of x‐ray beam transport. Figure 6 visually illustrates the formation of a concentrated electron hot spot at the center of the radiation field, as observed through film measurements. Raw films obtained in the presence of the MRI and MRI + ECD are shown in Figure 6a,b. Colormap enhanced Raw films obtained in the presence of the MRI and MRI + ECD are shown in Figure 6c, d. The intensity of the hot spot, as depicted on the film, appears to vary with the distance. The introduction of the ECD effectively mitigates the intensity of the hot spot. However, the ECD remains effective up to a certain distance, beyond which the intensity of the hot spot begins to increase again as shown in Figure 6.

### 3.2 MC simulations of dose slices and hot spot characterization

**FIGURE 7:**
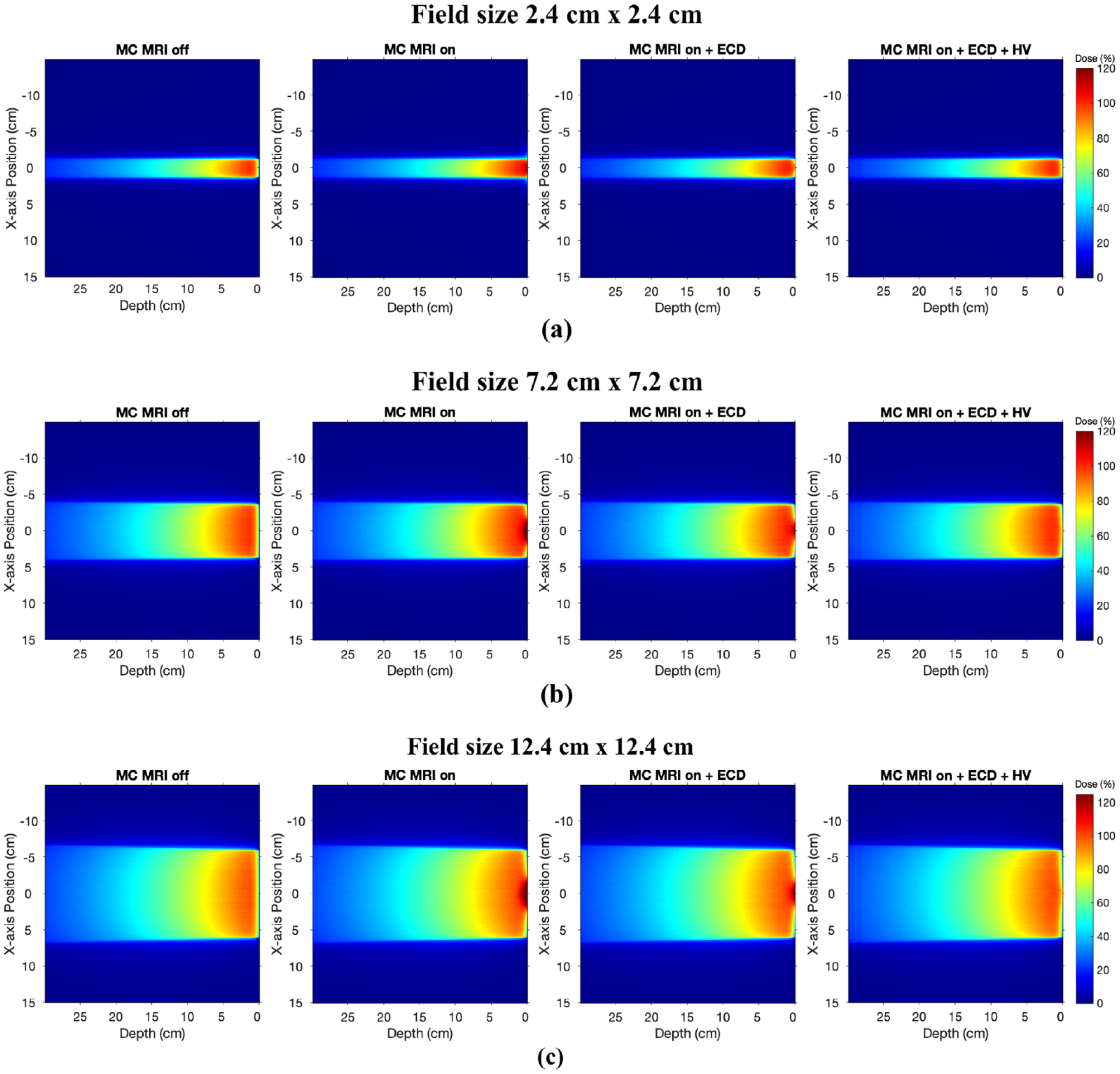
2D dose maps through the water phantom measured through MC simulations for field sizes (a) 2.4×2.4 ${\mathrm{cm}}^{2}$, (b) 7.2×7.2 ${\mathrm{cm}}^{2}$ and (c) 12.4×12.4 ${\mathrm{cm}}^{2}$. With MRI on, the focusing of electron contamination in the central axis of the beam can be seen clearly for all three field sizes. The addition of ECD purges the electron contamination to some extent, however, the combination of ECD and HV can effectively purge the focusing effect of electron contamination. ECD, electron contamination deflector; HV, helium gas volume; MC, Monte Carlo; MRI, magnetic resonance imaging.

**FIGURE 8:**
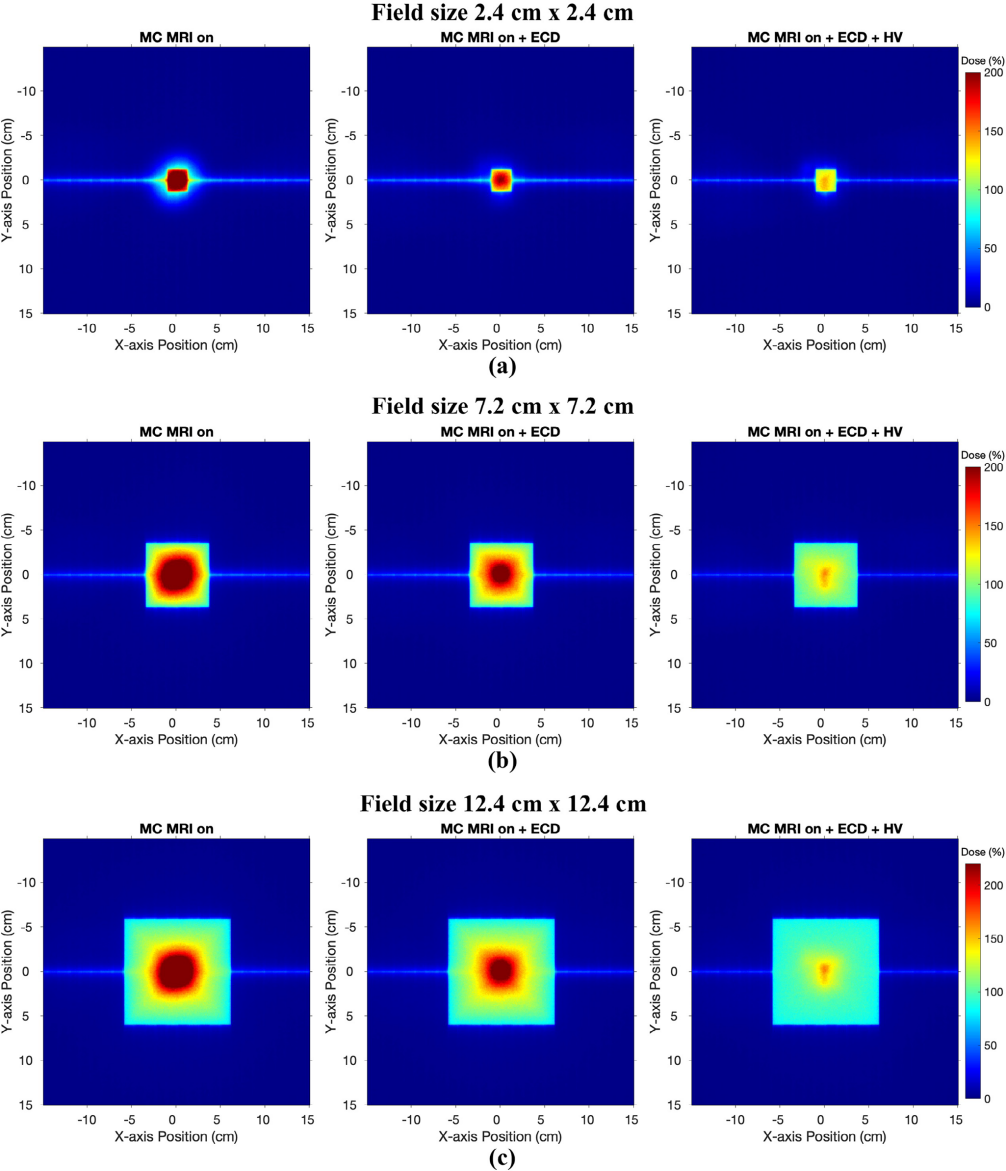
2D dose maps on the surface of the water phantom measured through MC simulations for field sizes (a) 2.4×2.4 ${\mathrm{cm}}^{2}$, (b) 7.2×7.2 ${\mathrm{cm}}^{2}$ and (c) 12.4×12.4 ${\mathrm{cm}}^{2}$. With MRI on, the hot spot can be seen clearly due to the electron focusing effect. The efficiency of ECD and HV is evident for all three field sizes as the hot spots shrink significantly for all three field sizes. ECD, electron contamination deflector; HV, helium gas volume; MC, Monte Carlo; MRI, magnetic resonance imaging.

Figure 7 represents a central 2D dose map through the water phantom with MC MRI off, MC MRI on, MC MRI on with ECD added, and MC MRI on with both ECD and the HV for all three field sizes. Figure 8 presents the 2D skin dose in the first 1 mm depth of the phantom for the three field sizes presented from left to right. The color maps are employed on the images to highlight the intensity of hot spots on the skin above the nominal dose at $d_{max}$ which is 100% dose at 1.5 cm depth for a 6 MV photon beam without the magnetic field (MC MRI off). These dose maps demonstrate how the dose hot spot is localized around the beam's central axis within the first mm of the phantom. The addition of the ECD reduces the intensity of the hot spot but the reduction is more effective and pronounced when combined with the HV. We also note the central thin horizontal line of dose observed in Figure 8 for all three field sizes. This corresponds to the MLC leaf tip transmission of the out‐of‐field closed leaf pairs.

**FIGURE 9:**
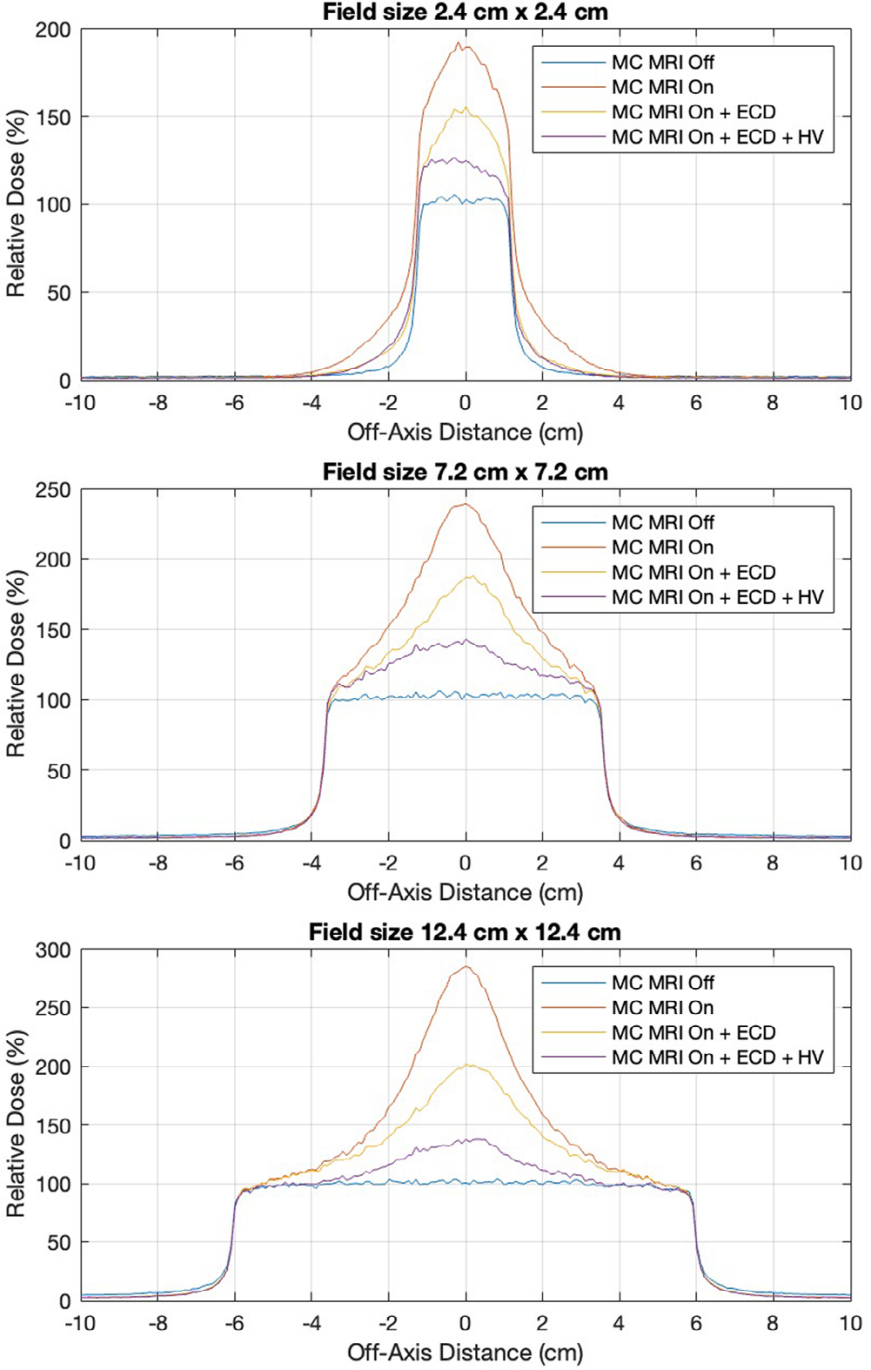
Beam profiles measured through MC simulations in the first 1 mm depth for field sizes of 2.4×2.4 ${\mathrm{cm}}^{2}$, 7.2×7.2 ${\mathrm{cm}}^{2}$ and 12.4×12.4 ${\mathrm{cm}}^{2}$. The effectiveness of the ECD and HV is demonstrated across all three field sizes. ECD, electron contamination deflector; HV, helium gas volume; MC, Monte Carlo.

Figure 9 displays surface dose (first 1 mm depth) beam profiles obtained from the MC simulations across all field sizes. This further demonstrates the effectiveness of the ECD and HV in reducing the skin dose. The profiles indicate the clear reduction in the intensity of the highly focused hot spot along the beam's central axis, as well as a reduction in the out‐of‐field dose.

### 3.3 Central axis PDDs and skin dose measurements at 70 μm depth

**FIGURE 10:**
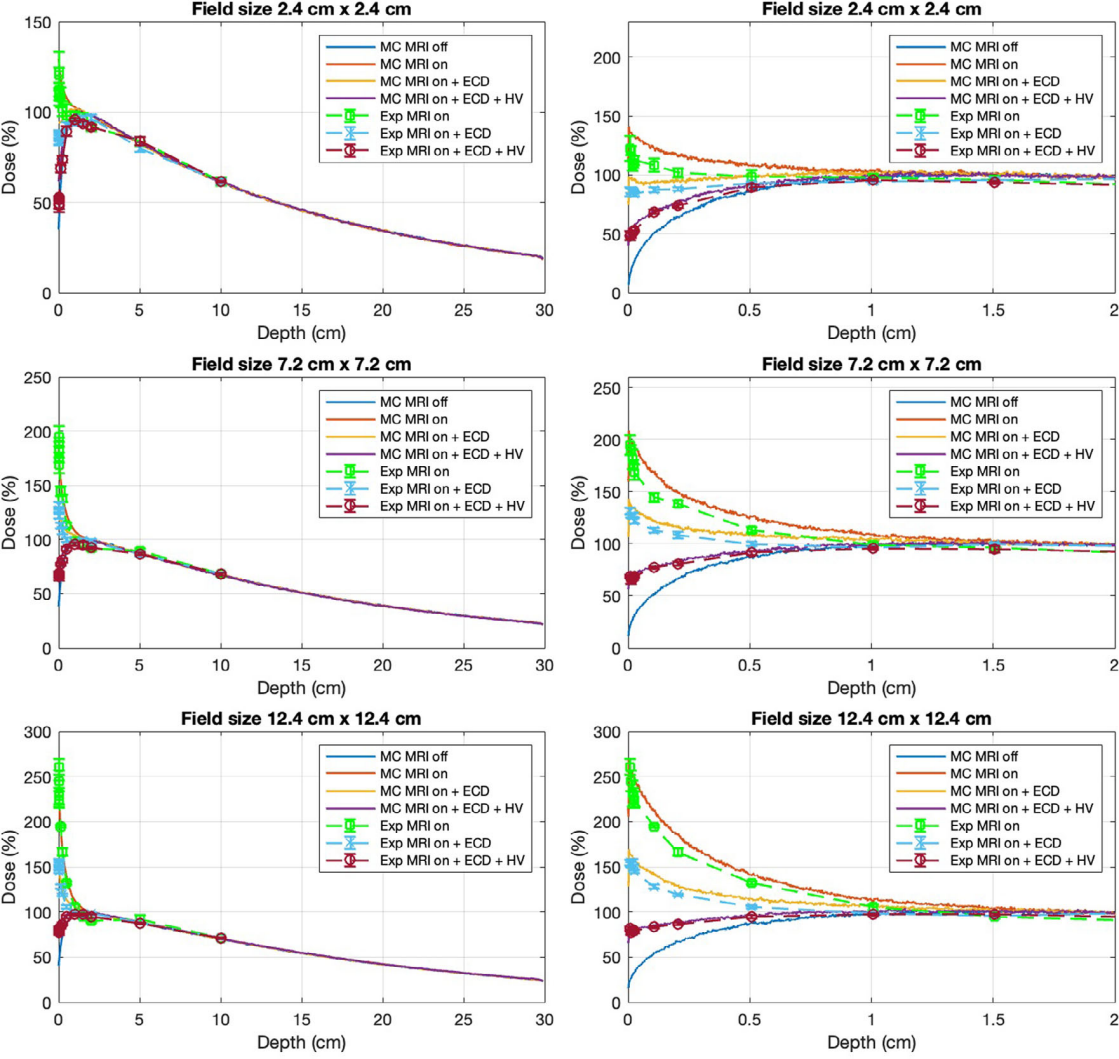
Percentage depth dose curves are shown for field sizes of 2.4×2.4 ${\mathrm{cm}}^{2}$, 7.2×7.2 ${\mathrm{cm}}^{2}$ and 12.4×12.4 ${\mathrm{cm}}^{2}$, based on both Exp measurements (Exp) and 3MC simulations (MC). On the left, the depth dose curves are shown up to 10 cm depth for Exp data and up to 30 cm depth for MC simulations. On the right, the first 2 cm depth of the curves are shown to highlight the steep dose gradient near the surface. The error bars from the Exp measurements are also added on the plots. MC, Monte Carlo; Exp, experimental.

Figure 10 presents the central axis percentage depth dose profiles for all three field sizes measured through MC and Exp. The plots on the left side show the depth dose distribution in the phantom, extending up to 10 cm for the Exp measurements and up to 30 cm for the MC simulations. On the right, the plots represent the dose in the first 2 cm of the phantom, measured using high‐resolution scoring voxels in the MC simulations. For comparison, the dose measured with MO*Skin*™ in the first 2 cm is also included in the same plots. Figure 9 shows the beam profiles measured through MC simulations for field sizes of 2.4×2.4 ${\mathrm{cm}}^{2}$, 7.2×7.2 ${\mathrm{cm}}^{2}$ and 12.4×12.4 ${\mathrm{cm}}^{2}$ at the first 1 mm depth of the phantom. Table 1 presents the skin dose values at a depth of 70 μm from the MC simulations and the Exp measurements. The Exp measurements had the maximum uncertainty of ±8.6%, while the MC simulations results had an uncertainty of approximately ±5%. The percentage of error between the MC simulations and the Exp values is approximately 11% for a 2.4×2.4 ${\mathrm{cm}}^{2}$ field size, 5.33% for a 7.2×7.2 ${\mathrm{cm}}^{2}$ field size, and 5.48% for a 12.4×12.4 ${\mathrm{cm}}^{2}$ field size.

**TABLE 1:** Skin dose values measured at a depth of 70μm. Exp measurements were conducted using the MO*Skin*™ detector, while MC simulation values were obtained using a high‐resolution dose scoring volume. The values are normalized at 10 cm depth and expressed as a percentage of the dose at $d_{max}$ (1.5 cm depth) for near 0 T simulations (MC MRI off).

Field Size at isocenter		Skin Dose (%) of $d_{max}$			
		MRI Off	MRI On	ECD	ECD + HV
2.4×2.4 ${\mathrm{cm}}^{2}$	Exp	N/A	122.5 ± 10.6	85.7 ± 3.9	48.3 ± 3.7
	MC	16.4 ± 0.8	135.3 ± 6.7	96.9 ± 4.8	54.6 ± 2.7
	Error (%)	N/A	9.4	11.4	11.6
7.2×7.2 ${\mathrm{cm}}^{2}$	Exp	N/A	194.8 ± 9.5	131.1 ± 3.2	68.6 ± 1.3
	MC	22.5 ± 1.2	199.6 ± 9.9	137.2 ± 6.8	72.5 ± 3.6
	Error (%)	N/A	2.4	4.4	5.3
12.4×12.4 ${\mathrm{cm}}^{2}$	Exp	N/A	260.4 ± 8.5	154.6 ± 2.9	81.5 ± 2.6
	MC	25.5 ± 1.3	258.4 ± 12.9	163.6 ± 8.2	82.4 ± 4.1
	Error (%)	N/A	0.8	5.5	1.1

Abbreviations: ECD, electron contamination deflector; Exp, experimental; HV, helium gas volume; MC, Monte Carlo; MRI, magnetic resonance imaging.

## 4 DISCUSSION

### 4.1 MC simulations of dose slices and hot spot characterization

The MC simulation framework provides a detailed analysis of the hot spots of skin dose in the Australian MRI‐linac prototype. According to the simulation results, the strong inline magnetic field influences the tracks of the secondary electrons produced within the beam. These electrons are highly focused within the center of the beam, causing a hot spot on the skin surface. The intensity of the hot spot increases for larger field sizes as electron contamination generated from the air and collimation system makes major contribution to the skin dose, resulting in the more concentrated and focused hot spot in the center of the beam. The influence of the magnetic field is also noticeable on the films as the hot spot becomes more intense with the increase in the distance from the source.

### 4.2 Electron contamination purging and skin dose values using the ECD and HV

The results obtained through MC simulations and Exp measurements demonstrate that placing an ECD on the proximal (or isocenter) side of the MLC can effectively minimize electron contamination and reduce overall skin dose. As predicted by the MC simulations and further validated by the Exp measurements, the presence of ECD prototype alone reduces the skin dose to 85.7% for a 2.4×2.4 ${\mathrm{cm}}^{2}$ field size, 131.1% for a 7.2×7.2 ${\mathrm{cm}}^{2}$ field size, and 154.6% for a 12.4×12.4 ${\mathrm{cm}}^{2}$ field size. However, these values are still relatively high, as the remaining leading cause of skin dose comes from air‐generated electrons between the ECD and the phantom surface. The qualitative analysis through the film measurements also shows that while the ECD reduces hot spot intensity up to a certain distance, the presence of the air column reintroduces the electrons. To eliminate air‐generated electron contamination, HV has been added between the ECD and MRI‐isocentre in the final stage of the simulations and subsequently in the experiments. The results from MC simulations in Figure 7 and Figure 8 demonstrate that using HV in the air space above the phantom surface can further reduce the skin dose significantly. The Exp measurements also show that in the presence of the HV along with ECD, the skin dose can be reduced to 48.3% for a 2.4×2.4 ${\mathrm{cm}}^{2}$ field size, 68.6% for a 7.2×7.2 ${\mathrm{cm}}^{2}$ and 81.5% for a 12.4×12.4 ${\mathrm{cm}}^{2}$. Thus, combining the ECD with a HV significantly reduces the intensity and cross‐sectional area of hot spots for all field sizes.

The efficiency of the helium volume may have been compromised to some extent due to the thin plastic layer of the bag, which could be acting as a source of secondary electrons. However, this layer is necessary for containing the helium gas and has been kept as minimal as practically possible.

Several skin dose reduction methods have been proposed in the literature in Section I, each with limitations. With an off‐axis irradiation, surface doses were reduced to 24.9 %, 39.2 %, and 47.3 % for 1.9× 1.9 ${\mathrm{cm}}^{2}$, 5.8× 5.8 ${\mathrm{cm}}^{2}$, and 9.7× 9.6 ${\mathrm{cm}}^{2}$ field sizes, respectively. However, the use of large and open field sizes is restricted with off‐axis irradiation, as the distance between the edge of the electron hot spot and the treatment field surpasses the physical constraints of the MRI system. Therefore, determining the optimal offset distance is crucial.^41^ Scattering foils are ineffective as they produce secondary electrons, which contribute to increased skin dose. A flattened beam provides no advantage, as higher‐energy Compton electrons can more easily reach the treatment isocenter compared to the current FFF beam. A 2 cm water bolus reduces skin dose along the central axis but increases surface dose outside this region in the inline MRI‐linac. The skin dose at 70 μm depth is field‐dependent, increasing with larger field sizes. Although the bolus effectively reduces skin dose for medium and large fields, its impact is minimal for small fields. Additionally, air gaps introduce electron contamination, requiring bolus optimization.^41^

The generally good agreement between the MC simulations and the Exp measurements using the MO*Skin*™ detector also demonstrates the accuracy of our Geant4 MC model. Although the differences exceed 10% for the 2.4×2.4 ${\mathrm{cm}}^{2}$ field, the overall trend shows that the match improves for larger field sizes. We note that there are minor misalignments in the MRI isocenter and beamline isocenter,^39^ so there will be some small differences between our “ideal” simulation setup and the actual MRI‐linac system. Any dosimetric implications would be most pronounced at the smaller field sizes, as the hot spot is comparable to the x‐ray field size. One method to explore this theory would be to simulate a non‐perfect MRI‐linac design by deliberately misaligning the MRI and radiation beam isocenter.

The results obtained in this study pave the way for future research and the potential integration of the ECD and HV prototypes into the Australian MRI‐linac system, with the goal of minimizing hot spots on the skin. This also underscores the efficiency and practicality of the MO*Skin*™ detector for skin dosimetry in MRI‐linac applications, as the detector has demonstrated a stable and linear response in the presence of the magnetic field. The methods used in this work can be readily applied to other inline MRI‐linac prototypes if skin dose increases are deemed problematic. The exact design and locations of the ECD and HV would need to be optimized for the particular MRI‐linac system being investigated.

## 5 CONCLUSION

Our study is the first to evaluate the effectiveness of an ECD and HV in reducing skin dose in a preclinical prototype of high‐field (1 T) inline MRI‐linac system. The findings demonstrate that these devices can decrease the skin dose hot spot below $d_{max}$ for field sizes up to 12.4×12.4 ${\mathrm{cm}}^{2}$ at the treatment isocenter, outperforming the conventional 2 cm surface bolus approach. These encouraging results support the integration of an ECD and HV prototype in future clinical setups, as well as the potential to be studied for application in other inline MRI‐linac prototypes.

## CONFLICT OF INTEREST STATEMENT

The authors declare no conflicts of interest.

## References

[1] Lagendijk JJW , Raaymakers BW , Berg dCATV , Moerland MA , Philippens ME , Vulpen vM . MR guidance in radiotherapy. Phys Med Biol. 2014;59(21):R349‐R369. 25322150 10.1088/0031-9155/59/21/R349

[2] Raaymakers BW , Jürgenliemk‐Schulz IM , Bol GH , et al. First patients treated with a 1.5 T MRI‐Linac: clinical proof of concept of a high‐precision, high‐field MRI guided radiotherapy treatment. Phys Med Biol. 2017;62(23):L41‐L50. 29135471 10.1088/1361-6560/aa9517

[3] Mutic S , Dempsey JF . The ViewRay System: Magnetic Resonance ‐ Guided and Controlled Radiotherapy. Semin Radiat Oncol. 2014;24(3):196‐199. 24931092 10.1016/j.semradonc.2014.02.008

[4] Raaymakers BW , Raaijmakers AJE , Kotte ANTJ , Jette D , Lagenddijk JJW . Integrating a MRI scanner with a 6 MV radiotherapy accelerator: dose deposition in a transverse magnetic field. Phys Med Biol. 2004;49:4109‐4118. 15470926 10.1088/0031-9155/49/17/019

[5] Raaijmakers AJE , Raaymakers BW , Lagendijk JJW . Integrating a MRI scanner with a 6 MV radiotherapy accelerator: dose increase at tissue‐air interfaces in a lateral magnetic field due to returning electrons. Phys Med Biol. 2005;50:1363‐1376. 15798329 10.1088/0031-9155/50/7/002

[6] Raaijmakers AJE , Raaymakers BW , Meer v . dS, Lagendijk JJW. Integrating a MRI scanner with a 6 MV radiotherapy accelerator: impact of the surface orientation on the entrance and exit dose due to the transverse magnetic field. Phys Med Biol. 2007;52:929‐939. 17264362 10.1088/0031-9155/52/4/005

[7] Raaijmakers AJE , Raaymakers BW , Lagendijk JJW . Experimental verification of magnetic field dose effects for the MRI‐accelerator. Phys Med Biol. 2007;52:4283‐4291. 17664608 10.1088/0031-9155/52/14/017

[8] Meijsing I , Raaymakers BW , Raaijmakers AJE , et al. Dosimetry for the MRI accelerator: the impact of a magnetic field on the response of a Farmer NE2571 ionization chamber. Phys Med Biol. 2009;54:2993‐3002. 19387100 10.1088/0031-9155/54/10/002

[9] Reynolds M , Fallone BG , Rathee S . Dose response of selected ion chambers in applied homogeneous transverse and longitudinal magnetic fields. Med Phys. 2013;40(4):042102. 23556912 10.1118/1.4794496

[10] Reynolds M , Fallone BG , Rathee S . Dose response of selected solid state detectors in applied homogeneous transverse and longitudinal magnetic fields. Med Phys. 2014;41(9):092103. 25186403 10.1118/1.4893276

[11] Raaijmakers AJE , Raaymakers BW , Lagendijk JJW . Magnetic‐field‐induced dose effects in MR‐guided radiotherapy systems: dependence on the magnetic field strength. Phys Med Biol. 2008;53(4):909‐923. 18263948 10.1088/0031-9155/53/4/006

[12] Kirkby C , Stanescu T , Rathee S , Carlone M , Murray B , Fallone BG . Patient dosimetry for hybrid MRI‐radiotherapy systems. Med Phys. 2008;35(3):1019‐1027. 18404937 10.1118/1.2839104

[13] Smit K , Asselen vB , Kok JGM , Aalbers AHL , Lagendijk JJW , Raaymakers BW . Towards reference dosimetry for the MR‐linac: magnetic field correction of the ionization chamber reading. Phys Med Biol. 2013;58(17):5945‐5957. 23938362 10.1088/0031-9155/58/17/5945

[14] Begg J , Jelen U , Moutrie Z , Oliver C , Holloway L , Brown R . ACPSEM position paper: dosimetry for magnetic resonance imaging linear accelerators. Phys Eng Sci Med. 2023;46:1‐17. 36806156 10.1007/s13246-023-01223-w PMC10030536

[15] Oborn BM , Metcalfe PE , Butson MJ , Rosenfeld AB . High resolution entry and exit monte carlo dose calculations from a linear accelerator 6MV beam under influence of transverse magnetic fields. Med Phys. 2009;36(8):3549‐3559. 19746789 10.1118/1.3157203

[16] Oborn BM , Metcalfe PE , Butson MJ , Rosenfeld AB . Monte Carlo characterization of skin doses in 6 MV transverse field MRI‐linac systems: Effect of field size, surface orientation, magnetic field strength, and exit bolus. Med Phys. 2010;37(10):5208‐5217. 21089754 10.1118/1.3488980

[17] Bielajew AF . The effect of strong longitudinal magnetic fields on dose deposition from electron and photon beams. Med Phys. 1993;20:1171‐1179. 8413027 10.1118/1.597149

[18] S Ramahi SN , Chu,. J. Achieving a smaller penumbra region for better planning in conformal radiotherapy by using a longitudinal magnetic field. Annu Int Conf IEEE Eng Med Biol Soc. 2000;22(4):3260‐3263.

[19] Naqvi SA , Li XA , Ramahi SW , Chu JC , Ye SJ . Reducing loss in lateral charged‐particle equilibrium due to air cavities present in x‐ray irradiated media by using longitudinal magnetic fields. Med Phys. 2001;28(4):603‐611. 11339758 10.1118/1.1357816

[20] Weinhous MS , Nath R , Schulz RJ . Enhancement of electron beam dose distributions by longitudinal magnetic fields: Monte Carlo simulations and magnet system optimization. Med Phys. 1985;12:598‐603. 4046994 10.1118/1.595681

[21] Litzenberg DW , Fraass BA , McShan DL , et al. An apparatus for applying strong longitudinal magnetic fields to clinical photon and electron beams. Phys Med Biol. 2001;46:N105‐N115. 11384072 10.1088/0031-9155/46/5/401

[22] Chen Y , Bielajew AF , Litzenberg DW , Moran JM , Becchetti FD . Magnetic confinement of electron and photon radiotherapy dose: A Monte Carlo simulation with a nonuniform longitudinal magnetic field. Med Phys. 2005;32(12):3810‐3818. 16475781 10.1118/1.2011091

[23] Fallone B , Murray B , Rathee S , et al. First MR images obtained during megavoltage photon irradiation from a prototype integrated linac‐MR system. Med Phys. 2009;36:2084. 19610297 10.1118/1.3125662

[24] Fallone BG . The rotating biplanar linac ‐ magnetic resonance imaging system. Semin Radiat Oncol. 2014;24(3):200‐202. 24931093 10.1016/j.semradonc.2014.02.011

[25] Petti PL , Goodman MS , Gabriel TA , Mohan R . Investigation of build‐up dose from electron contamination of a clinical photon beams. Med Phys. 1983;10:18‐24. 6405143 10.1118/1.595287

[26] Ahnesjo A , Andreo P . Determination of effective bremsstralung spectra and electron contamination for photon dose calculations. Phys Med Biol. 1989;34:1451‐1464. 2813512 10.1088/0031-9155/34/10/008

[27] Padikal TN , Deye JA . Electron contamination of a high‐energy x‐ray beam. Phys Med Biol. 1978;23:1086‐1092. 733897 10.1088/0031-9155/23/6/004

[28] Nilsson B , Brahme A . Absorbed dose from secondary electrons in high energy photon beams. Phys Med Biol. 1979;24:901‐912. 117462 10.1088/0031-9155/24/5/003

[29] Biggs PJ , Russell MD . An investigation into the presence of secondary electrons in megavoltage photon beams. Phys Med Biol. 1983;28:1033‐1043. 6415682 10.1088/0031-9155/28/9/003

[30] Petti PL , Goodman MS , Sisterson JS , Biggs PJ , Gabriel TA , Mohan R . Sources of electron contamination for the Clinac‐35 25MV photon beam. Med Phys. 1983;10:856‐861. 6419032 10.1118/1.595348

[31] Yorke ED , Ling CC , Rustgi S . Air‐generated electron contamination of 4 and 10MV photon beams: a comparison of theory and experiment. Phys Med Biol. 1985;30:1305‐1314. 4089019 10.1088/0031-9155/30/12/004

[32] Sjogren R , Karlsson M . Electron contamination in high energy photon beams. Med Phys. 1996;23(11):1873‐1881. 8947901 10.1118/1.597750

[33] Zhu TC , Palta JR . Electron contamination in 8 and 18 MV photon beams. Med Phys. 1997;25(1):12‐19. 10.1118/1.598169 9472821

[34] Butson MJ , Cheung T , Yu P , Metcalfe PE . Simulation and measurement of air generated electron contamination in radiotherapy. Radiat. Meas. 2000;32:105‐111.

[35] Medina AL , Teijeiro A , Garcia J , et al. Characterization of electron contamination in megavoltage photon beams. Med Phys. 2005;32(5):1281‐1292. 15984680 10.1118/1.1895793

[36] Oborn B , Metcalfe P , Butson M , Rosenfeld A , Keall P . Electron contamination modeling and skin dose in 6 MV longitudinal field MRIgRT: Impact of the MRI and MRI fringe field. Med Phys. 2012;39(2):874‐890. 22320797 10.1118/1.3676181

[37] Liney GP , Dong B , Begg J , et al. Technical Note: experimental results from a prototype high‐field inline MRI‐linac. Med Phys. 2016;43(9):5188‐5194. 27587049 10.1118/1.4961395

[38] Oborn BM , Kolling S , Metcalfe PE , Crozier S , Litzenberg DW , Keall PJ . Electron contamination modeling and reduction in a 1 T open bore inline MRI‐linac system. Med Phys. 2014;41(5):051708. 24784374 10.1118/1.4871618

[39] Begg J , Alnaghy SJ , Causer T , et al. Technical note: experimental characterization of the dose deposition in parallel MRI‐linacs at various magnetic field strengths. Med Phys. 2019;46(11):5152‐5158. 31419317 10.1002/mp.13767

[40] Roberts NF , Patterson E , Jelen U , et al. Experimental characterization of magnetically focused electron contamination at the surface of a high‐field inline MRI‐linac. Med Phys. 2019;46(12):5780‐5789. 31633212 10.1002/mp.13847

[41] Patterson E , Oborn BM , Cutajar D , et al. Characterizing magnetically focused contamination electrons by off‐axis irradiation on an inline MRI‐Linac. J App Clinic Med Phys. 2022;23(6):e13591. 10.1002/acm2.13591 PMC9195023 35333000

[42] Tai M , Patterson E , Metcalfe PE , Rosenfeld A , Oborn BM . Skin dose modelling and measurement in a high field in‐line MRI‐Linac system. Front Phys. 2022;10. doi:10.3389/fphy.2022.902744

[43] Lee CS , Yoo MJ , Yum HY . Reduction of electron contamination using a filter for 6MV photon beam. J Korean Soc Ther Radiol Oncol. 1997;15(2):159‐165.

[44] Jelen U , Dong B , Begg J , et al. Dosimetric optimization and commissioning of a high field inline MRI‐Linac. Front Oncol. 2020;10:136. 32117776 10.3389/fonc.2020.00136 PMC7033562

[45] Shan S , Liney GP , Tang F , et al. Geometric distortion characterization and correction for the 1.0 T Australian MRI‐linac system using an inverse electromagnetic method. Med Phys. 2020;47(3):1126‐1138. 31856301 10.1002/mp.13979

[46] Butson MJ , Cheung T , Yu P , Metcalfe PE . Evaluation of a radiotherapy electron contamination deflecting system. Radiat Meas. 2000;32:101‐104.

[47] Jong WL , Wong JHD , Ung NM , et al. Characterization of MOSkin detector for in vivo skin dose measurement during megavoltage radiotherapy. J Appl Clin Med Phys. 2014;15:120‐132. 10.1120/jacmp.v15i5.4869 PMC5711095 25207573

[48] COMSOL. *COMSOL (Multphysics)*. Stockholm, Sweden: COMSOL (2021).

[49] ICRP . Recommendations of the ICRP. ICRP Publication 26. Ann. ICRP 1 (3), 1977.

[50] International Commission on Radiation Units and Measurements. “Determination of dose equivalents from external radiation sources: Part 2.”, 1988.

[51] Kwan I , Rosenfeld A , Qi Z , et al. Skin dosimetry with new MOSFET detectors. Radiat Meas. 2008;43:929‐932.

